# Toward Setting Minimum and Optimal Data to Report for Malaria Molecular Surveillance with Targeted Sequencing: The “What” and “Why”

**DOI:** 10.4269/ajtmh.25-0147

**Published:** 2025-09-25

**Authors:** Jonathan J. Juliano, Cecile P. G. Meier-Scherling, Neeva Wernsman Young, George A. Tollefson, Sean V. Connelly, Jonathan B. Parr, Melissa D. Conrad, Jacob M. Sadler, Christopher M. Hennelly, Ashenafi Assefa, Lucy Okell, Abebe A. Fola, Karamoko Niaré, Jacob Marglous, Kelly Carey-Ewend, Ronald Futila Kyong-Shin, Isabela Gerdes Gyuricza, Gina Cuomo-Dannenburg, Shazia Ruybal-Pesántez, Oliver J. Watson, Robert Verity, Jeffrey A. Bailey

**Affiliations:** ^1^ Division of Infectious Diseases, Department of Medicine, School of Medicine, University of North Carolina, Chapel Hill, North Carolina;; ^2^ Institute for Global Health and Infectious Diseases, University of North Carolina, Chapel Hill, North Carolina;; ^3^ Department of Epidemiology, Gillings School of Global Public Health, University of North Carolina, Chapel Hill, North Carolina;; ^4^ Curriculum in Genetics and Molecular Biology, University of North Carolina, Chapel Hill, North Carolina;; ^5^ Center for Computational Molecular Biology, Brown University, Providence, Rhode Island;; ^6^ Department of Pathology and Laboratory Medicine, Brown University, Providence, Rhode Island;; ^7^ MD-PhD Program, School of Medicine, University of North Carolina, Chapel Hill, North Carolina;; ^8^ Department of Medicine, University of California- San Francisco, San Francisco, California;; ^9^ Ethiopian Public Health Institute, Addis Ababa, Ethiopia;; ^10^ MRC Centre for Global Infectious Disease Analysis, School of Public Health, Imperial College, London, United Kingdom;; ^11^ Curriculum in Bioinformatics and Computational Biology, University of North Carolina, Chapel Hill, North Carolina;; ^12^ National Institute of Biomedical Research, Kinshasa, Democratic Republic of the Congo;; ^13^ Instituto de Microbiología, Universidad San Francisco de Quito, Ecuador

## Abstract

The coronavirus disease 2019 pandemic showcased the power of genomic surveillance in tracking infectious diseases, driving rapid public health responses, and global collaboration. This same infrastructure is being leveraged for malaria molecular surveillance (MMS) in Africa to address challenges such as artemisinin partial resistance and deletions in the *Plasmodium falciparum *histidine-rich protein 2 and 3 genes. However, variability in reporting sequencing methods and data reporting currently limits the validation, comparability, and reuse of data. To maximize the impact of MMS, minimal and optimal data that are key for validation and maximizing transparency and findable, accessible, interoperable, and reusable principles are proposed for reporting. Rather than focusing on specific data formats, in the current study, the authors propose what should be reported and why. Progressing to reporting individual infection-level polymorphism or microhaplotype data is central to maximizing the impact of MMS. Reporting must adhere to local regulatory practices and ensure proper data oversight and management, preventing data colonialism and preserving opportunities for data generators. With malaria’s challenges transcending borders, reporting and adopting standardized practices are essential to advancing research and strengthening global public health efforts.

## INTRODUCTION

Pathogens evolve through mutations that can modify their virulence, transmissibility, and response to interventions. These evolutionary changes drive dynamic processes with consequences at regional, national, and global scales. During the coronavirus disease 2019 (COVID-19) pandemic, molecular surveillance played a central role in facilitating responses to rapidly evolving strains. Large-scale sequencing of severe acute respiratory syndrome coronavirus 2 (SARS-CoV-2) variants provided the ability to quickly identify new variants, allowing the public health community to swiftly monitor their spread and develop and deploy new interventions, such as updating vaccines.^1^^,^^2^ Moreover, the ability to study transmission using sequencing enabled a better understanding of the risks posed by infected individuals to uninfected populations.^3^^–^^7^ The terms “sequencing” and “variant” became commonplace in the public’s vocabulary, and an appreciation for the impact of these tools broadened. Community efforts, such as NextStrain, enabled interactive, close-to-real-time monitoring of pathogen spread by tracking genetic variation and evolution across broad geographic areas, combined with detailed information on the locations where isolates were collected.^8^ Modeling groups from around the globe were able to leverage this information to provide robust inference of epidemiological dynamics, inform outbreak response, help predict the eventual impact of the pandemic, and infer undetected infections via seropositivity.^9^^–^^12^ There were challenges associated with genomic surveillance of COVID-19 during the pandemic, including differences in initial methods and quality control, inconsistencies in clinical and demographic data reporting, delays in sharing, and slow compilation, which limited the utility of comprehensive datasets.^13^ Since then, the WHO’s Global Strategy for Genomic Surveillance of Pathogens with Pandemic and Epidemic Potential, 2022–2032, has recommended that pathogen genomic surveillance be introduced in every country of the world.^14^ In addition, newly established networks, such as the WHO International Pathogen Surveillance Network (https://www.who.int/initiatives/international-pathogen-surveillance-network) and the Africa Pathogen Genomics Surveillance Network (https://africacdc.org/priority-pathogens-and-use-cases-for-genomic-surveillance-in-africa/), are advocating for the use of pathogen genomics to inform public health decision-making.

### Molecular surveillance has now become integral to understanding multiple pathogens of global public health significance, including malaria.

Malaria is an endemic disease across the world, particularly in Africa, where the vast majority of cases and approximately half a million deaths occur annually. Over the last 5 years, a significant investment has been made in malaria molecular surveillance (MMS) by foundations such as the Gates Foundation, public health agencies including the Africa CDC and the US CDC, and research agencies, such as the United States NIH, Japan International Cooperation Agency, and European and Developing Countries Clinical Trials Partnership, as well as through use of Global Fund support to national malaria control programs and via direct investment by countries. Molecular and genomic surveillance of malaria will impact both public health decisions and the fundamental understanding of the parasite’s biology. Multiple use cases for molecular surveillance have been outlined previously, including:^15^

• Identifying the molecular mechanism or origin of drug and diagnostic resistance;
• Monitoring the prevalence, frequency, and spread of drug or diagnostic resistance markers;
• Classifying outcomes in therapeutic efficacy studies (TESs) as reinfection, recrudescence, or, in the case of *Plasmodium vivax,* relapse;
• Estimating transmission intensity;
• Estimating the connectivity and movement of parasites between geographically distinct populations;
• Classifying malaria cases as locally acquired or imported from another population;
• Reconstructing granular patterns of transmission.

### The effective use of MMS is challenged by multiple issues.

One central challenge is the timeliness of results being communicated to partners who enact policies, such as National Malaria Control Programs and the WHO. There are multiple reasons for this: 1) the complex nature of datasets can lead to long analysis times, 2) data may be held for conferences or high-impact manuscripts, or 3) data may not be generated in-country or involve key stakeholders. This highlights the importance of “How” MMS is performed and by “Who.” There is an ongoing effort to facilitate the transition of African institutions, including government institutions, to conduct the work and manage the data. This process is essential for ensuring that MMS has the greatest possible impact because early data sharing can have direct effects. An example of the impact of early data sharing and academic production is highlighted by the recent co-publication of an article in *Lancet Microbe* and *Lancet Infectious Diseases* about artemisinin partial resistance (ART-R) in Tanzania. In 2024, we reported on the prevalence of ART-R in Tanzania from 2021.^16^ Although these data took time to reach the broader academic audience, nearly 3 years after collection, it had been shared with the Tanzania National Malaria Control Program (NMCP) and in-country WHO representatives. This led to a TES, which was published by Ishengoma et al. at approximately the same time.^17^ This robust interaction between surveillance and evaluation occurred because the primary driver of this work was a strong collaboration of officials from the Ministry of Health and MMS experts (local and international) under the leadership of the National Institute for Medical Research in Tanzania. Answering the questions of “How is MMS conducted” and “Who is conducting it” presents its own challenges; however, the current perspective is focused on two different questions: the “What” in terms of data presentation and the “Why” of the selection of the data to be shared.

### Malaria genomics is challenging given the complexity of the pathogen and the infection.

Malaria genomics has blossomed with the emergence of next-generation sequencing. Similar to COVID-19, increased sequencing capabilities have enabled unprecedented insight into parasite genetics and significantly improved the ability to monitor emerging threats, such as drug and diagnostic resistance. However, compared with viral genomics, *Plasmodium spp.* (and other eukaryotic pathogens) present differing and more numerous challenges. This includes a high prevalence of infections with low parasite densities, where samples are overwhelmingly made up of human host DNA. Low parasitemia samples are often challenging to sequence and require enrichment techniques to capture enough parasite genomic material.^18^ Mixed infections with minor strains or multiple species that can occur at low relative abundances limit their detection relative to the sequencing error rate.^19^ Additionally, eukaryotic recombination continually reassorts parts of the parasite genome, obfuscating relationships between parasites and making tracking transmission chains or parasite origins difficult. Beyond the complexities of biology, standardizing methods is challenging. Unlike SARS-CoV-2, it is not practical to constantly sequence the entire genome. Although *Plasmodium spp.* genomes are actually smaller relative to other eukaryotes, at only 23 megabases for *Plasmodium falciparum* (*P. falciparum*), their size still presents challenges related to the cost-effectiveness of leveraging only whole-genome sequencing (WGS) for surveillance. The cost of WGS remains prohibitively expensive for large-scale MMS, and instead, less expensive targeted sequencing approaches, such as multiplexed amplicon deep sequencing or molecular inversion probes (MIPs),^20^^–^^26^ are often used. Targeted sequencing methods are also more sensitive and better suited for samples with low parasitemia.^27^ To date, compared with WGS, bioinformatic tools have been relatively ad hoc and optimized for specific questions of interest.^19^^,^^28^^–^^30^ Therefore, a major challenge with targeted MMS is the heterogeneity in assays and analyses, as well as the need to ensure that data can be properly validated, combined, and rigorously analyzed.

All next-generation sequencing targeted and whole-genome approaches used for MMS essentially generate the same deep sequencing data. Therefore, the data reported can be highly similar regardless of the approach. Additionally, there are universal metrics that impart measures of read quality, coverage, and genotyping calls. Data reporting formats and standards for genetic information can be applied across single-target amplicon deep sequencing,^31^ simple amplicon panels,^20^ highly complex amplicon panels,^23^ and MIPs, as well as WGS.^25^ However, these guidelines cannot be fully applied to some older methods used for MMS, like microsatellites and Sanger sequencing.

### Malaria molecular surveillance is poised to help address urgent emerging challenges.

Malaria control programs in Africa face two threats that MMS will be particularly well-suited to address. First, the emergence and spread of ART-R is a growing major public health challenge. Since its first report in 2014 in Rwanda, mutations in the *P. falciparum* kelch13 (K13) protein associated with ART-R have been detected across multiple African countries. Multiple WHO-candidate and validated ART-R markers have been found along the Rift Valley, stretching from Eritrea to Rwanda.^32^^–^^34^ These mutations have reached a prevalence of more than 20% in many areas.^32^^–^^34^ Kelch13 polymorphisms have also been sporadically found across Africa in other locations and are now appearing in Southern Africa.^35^ However, the malaria community is currently in an advantageous position compared with that during the emergence of resistance to previous antimalarials. Unlike before, ART-R resistance markers have been identified before partner drugs co-formulated with artemisinin derivatives start to fail. Therefore, MMS can be leveraged to 1) evaluate and monitor the spread of resistance based on the K13 mechanism; 2) study parasite evolution and fitness associated with K13 mutations and those associated with artemisinin combination therapies (ACTs); and 3) directly monitor the impact of interventions put in place to reduce the spread of ART-R and preserve the efficacy of ACTs. Furthermore, genomics, particularly targeted deep sequencing, has the ability to provide “molecular correction” in TESs by distinguishing new infections from recrudescences (treatment failures) to determine if parasites found before and after treatment are the same strain.^30^^,^^36^ The second challenge is the emergence of “diagnostic-resistant” parasites in the Horn of Africa, where parasites exhibit a deletion of the genes that encode histidine-rich proteins (*hrps*) 2 and 3. Histidine-rich protein 2 and its paralog HRP3 are the primary antigens detected by *P. falciparum* malaria rapid diagnostic tests (RDTs) in Africa. These parasites, which lack the genes that encode these proteins, are not detected by widely used RDTs. They are spreading and have risen to high prevalence in the region, causing malaria control programs in countries like Eritrea and Ethiopia to progress toward alternative diagnostics.^37^^–^^40^

### How are MMS data currently being collated?

Current efforts to collate data on molecular threats for malaria control primarily occur through three mechanisms. The WorldWide Antimalarial Resistance Network (www.wwarn.org), formed in 2009, has been actively drawing from the published literature and collecting data through direct collaboration with investigators to populate interactive antimalarial resistance mapping tools. Although these visualizations and the underlying data have been a valuable resource, the timely integration of data from large genomic epidemiology studies has lagged behind. This is partly due to the labor-intensive nature of manual extraction and the lack of consistency or adequacy in the reported data. The WHO also maintains the Malaria Threats Map and interactive dashboards (https://apps.who.int/malaria/maps/threats/) for both antimalarial resistance and *hrp2* and *hrp3* gene deletion. Similar challenges exist for these tools and the underlying data, particularly with respect to the timely integration of data. Lastly, individual projects or national malaria control programs have developed their own dashboards to collate and visualize data. Given the potential benefits of combined data analysis, making the collation of data easier is critical for maximizing and streamlining its use.

### Focusing on what data should be reported both minimally and optimally for MMS.

Despite the rise in popularity of high-throughput sequencing, the malaria community has not addressed what key data need to be reported to properly leverage this information for control and elimination programmatic priorities and further scientific investigation. This lack of clarity has led to the haphazard and ambiguous publication of data or the limited release of the underlying data, which lessens their impact. In the present study, the focus is on the “what” and “why” of data reporting rather than the “how,” avoiding the second (and often unintentionally intertwined) step regarding data format and storage that often complicates these discussions. The goal is to recommend data reporting standards, recognizing that individual data generators must abide by local regulatory and data management requirements. In addition, different data generators may have varying resources, either financial or personnel, available to support data deposition. Currently, no data generator is meeting these standards on a routine basis, including the authors. These perspectives and proposed standards are the vision of the authors, as researchers who have worked extensively on MMS and with multiple partners, and are not intended to represent the views of NMCPs or the WHO. Beyond basic reporting, the authors believed that the proposed standards would be beneficial to the scientific community, ensuring rigorous findings and facilitating the broader use of the data. These standards are intended to set an “initial goal post” that the community can more broadly refine and move toward together. The requirements for depositing MMS data into repositories to ensure rigor, reproducibility, and reuse are highlighted in Table 1. The proposed reporting for sequencing data and metadata to allow for more rapid reuse toward MMS goals, at both the national and regional level, as well as for academic purposes to help advance our understanding of malaria, is highlighted in Table 2. It is worth noting that several common metrics used in MMS, such as multiplicity of infection and complexity of infection, are commonly used in data analysis. Reporting these metrics is obviously good practice, but they were not included in the minimal standards for reporting. This omission is partly because comparing across studies using different methods to assess these metrics may not be the most effective approach; rather, making the data available in a way that allows for the joint calculation of these metrics across studies would be more robust.

**Table 1:** Public repository deposition for rigor, reproducibility, and reuse

Variable	Minimum Standard	Optimal Standard
	Study and Participant Metadata	
Raw sequence	All studies should provide underlying raw sequencing data for reproducibility of findings by others.	Same as minimum.
	Raw sequencing data are the key to the true reproducibility and validity of any study and should be required. Without raw data, inappropriate analyses leading to called variants or microhaplotypes can never be properly addressed. This also optimizes data for use in other scientific questions.	
Metadata	All key variables, as deemed deidentified, used in the study for the published work deposited in a sustainable, uncontrolled public database (e.g., open access).	All key variables deposited in a public controlled database that enables full reanalysis and validation of the study, deposited in a sustainable, uncontrolled public database (e.g., access needs approval, as it may contain identifiable data)
	Full metadata can potentially lead to participant identification, although the risk of negative impact on study participants is low, given that malaria is a common, unstigmatized disease. Optimally, all data, exactly as used in published analyses, are deposited into a controlled database that allows for registered, vetted scientists to reproduce, validate, and extend work.	
Methods/code	Detailed methods used for processing sequence data, programs, settings, filtering, and analysis with metadata.	Fully reproducible coding pipeline that takes data and produces all results and figures from the main analysis.
	Although detailed written methods are key, for analysis, the exact code used to analyze data and generate figures allows others to examine and check methods. Deposition of code in GitHub (GitHub Inc., San Francisco, CA) or a similar platform is obligatory. New developing methods for code reproducibility, such as Code Ocean compute capsules (Code Ocean, New York, NY), are being implemented.^41^	
Sequencing panel/assay	Genomic locations sequenced and genotyped.	Complete description of panel target regions and any filtered regions that may have been ignored due to high levels of known sequencing errors.
	Understanding the gene or genomic locations assayed by a panel allows for better integration of data. Panel design should be deposited in an easily accessible public database that is fixed in a version at the time of the study. Combined with microhaplotype or allele depth, this allows for the retrospective determination of reference genotypes for new mutations found later, since the older study would have only revealed wild-type genotypes and thus not have indicated a nonvariant site. Filtered regions removed due to difficult-to-assess repeats or error-prone sequences are important because the underlying variation found in subsequent studies in these regions would require reanalysis. Microhaplotypes and their within-sample counts represent a compact format that is lossless and easily encodes how a missing mutation in earlier samples sets was missed.	
Controls	Set of parasite standards to provide context of sensitivity and specificity; all studies should be run with negative controls (e.g., human DNA or water).	In addition to controls, random replicate samples to assess assay variation in 5–10% of samples.
	Laboratory-derived controls ensure consistent assay performance but cannot address the sample quality for a given experiment. Thus, repeating a percentage of samples (biological replicates) and assays (technical replicates) provides a more robust assessment of a given sample set. Ultimately, replicates (duplicates or even triplicates) can help control for noise and jackpot events, although these efforts increase costs.	

**Table 2:** Specific metadata, variant data, measures, and statistics for malaria molecular surveillance

Variable	Minimum Standard	Optimal Standard
Study and Participant Metadata		
Date of collection	Month and year of collection; start and end date of study (maximal aggregation over a year).	Individual collection date (jittered if malaria diagnosis date is considered identifying information to maintain longitudinal order at site).
Location of collection	Collection site or aggregated neighboring collection sites with the GPS coordinate of the clinic used or the centroid of the neighborhood. Clinic, village, or town should be easily attainable.	Highest-resolution data possible (GPS location of household, clinic of collection, or town or city of collection) at the individual level (jittered if considered potentially identifying information).
Age at time of collection	Age in years at time of collection.	Age in years and months or years to a single decimal place (at study start if longitudinal).
Sex	As collected in the study.	As collected in the study.
Treatment status	Pretreatment or post-treatment. Important to understand if the frequencies or prevalences of drug resistance mutations could be skewed due to recent drug pressure in the individual.	Complicated studies with multiple time points should delineate the timing of sampling (e.g., TES).
Sampling strategy	Symptomatic, asymptomatic, community, clinic, etc. on a study level.	Assigned to each individual sample in cases of complex study design.
Travel information	If available.	Provide all travel information available at the individual level.
Sequencing and Genotyping Data		
Variant/ haplotype calls	Nucleotide or amino acid change at variant sites is called. Heterozygous or homozygous calls of known public health import. Individual-level data should be reported for specific mutations, including validated resistance variants without observed variant genotypes. Findable, accessible, interoperable, and reusable-format variant calls, such as VCF, or preferably GVCF, provided supplementally. With NGS data, reporting within-sample allele frequencies is important. These data should be unfiltered (no sites or samples removed beyond baseline initial variant e calling.	Individual-level full microhaplotypes if generated and genotyping data (amino acid and nucleotide, indels, etc.) across all regions sequenced and variants called and provided in FAIR formats. Development of microhaplotypes that maintain linkage information and are optimal. Microhaplotypes and, to a slightly lesser extent, full GVCFs allow for the examination of potential new mutations that might be captured but otherwise not recognized initially. These data should be unfiltered (no sites or samples removed beyond baseline initial variant e calling.
Read or UMI depth	Number of reads or UMIs informing each genotype (SNP or combination of SNPs) reported at each locus by individual. Total number of reads per locus reported. This is key to any quality assessment to determine how much weight each sample receives.	Number of reads or UMIs informing each full haplotype called (not just those reported in the manuscript) at each locus by individual. Total number of reads per locus reported. Read depth provides a limited approximation of the information content, whereas UMIs provide a more complete accounting traceable to individual molecules of the template in the sample.
Frequency (population and within-sample of allele or variant)	Average allele frequency for an aggregate site or region.	Within-sample allele frequencies for each participant; these can be calculated directly or from read depth/UMI counts.
	Allele frequency is not always reported compared with prevalence. However, frequency is much more robust for assessing sequencing error or low-level contamination. For instance, assume that in 100 samples, there are 10 samples with errors reporting K13 C580Y at a within-sample allele frequency of 1% each. Those 100 samples would result in a reported prevalence of 10% but only an average population allele frequency for 580Y of 0.1%. There is concern that such errors occur when there is a high percentage of mixed infections for a given mutation.	

FAIR = findable, accessible, interoperable, and reusable; GVCF = genomic variant call format; K13 = kelch13 NGS = next-generation sequencing; SNP = single-nucleotide polymorphism; TES = therapeutic efficacy study; UMI = unique molecular identifier; VCF = variant call format.

### The ultimate goal should be the deposition of deidentified individual participant sequence data and metadata into the public realm.

Findable, accessible, interoperable, and reusable (FAIR) principles should be followed to the greatest extent possible to allow for the most extensive use of data for other scientific questions.^42^^–^^44^ The lack of such data inhibits the forward advance of science, which prompted the US NIH to require this reporting standard for grants submitted after 2023. However, many publications still make data available upon request, which often results in delays in access and additional unstated requirements, such as authorship stipulations by the data holders. Malaria molecular surveillance studies supported by other funders or conducted by government agencies may not be mandated to deposit raw data in a similar manner.

### To maximize reproducibility and reuse, both raw sequencing data and initial variant calls should be shared with the community.

Sharing raw sequencing data at the individual participant level would allow for the fullest reuse and should be the gold standard for data sharing. However, full computational reanalysis would be laborious and costly, limiting its reuse by many groups. Therefore, reporting initial variant data with publication would enable the community to fully leverage previous sequencing data analysis to integrate across datasets. Although some initial filtering for quality, depth, or missingness may be part and parcel of variant or microhaplotype calling, providing baseline calls before applying more stringent filtering that may be needed for subanalyses is crucial. This initial call data allows others to reproduce results without downloading and recalling variations, and enables appropriate filtering for any additional analyses. Data on read depth per amplicon and raw numbers of reads supporting each allele variant are necessary. This is particularly important in maintaining the within-sample variant frequencies in cases of mixed infections. In addition, if available, data on both individual simple variants (e.g., single-nucleotide polymorphisms [SNPs] and small indels) and overall microhaplotypes (a segment of DNA containing two or more mutations) should be reported. Provision of the exact microhaplotypes (the full sequence or all variation from a single amplicon) is the optimal format for polymerase chain reaction (PCR)-based targeted sequencing because it preserves the observed linkage between polymorphisms that can contain important information, including the level of resistance, origin of a mutation, and spatial or relatedness information that might otherwise be lost. Some examples include *P. falciparum* dihydrofolate reductase, *P. falciparum* dihydropteroate synthase, *P. falciparum* chloroquine resistance transporter, and *P. falciparum* multidrug resistance protein 1.^45^^–^^49^ Similarly, copy number measures, whether from sequence data or other methods (e.g., quantitative real time polymerase chain reaction [qPCR]), should be reported as a continuous copy number for the sample, not a rounded value, allowing for the further assessment of overall variance. It is essential that variant reporting also meets FAIR requirements.^44^ Reporting the underlying read depth for microhaplotypes in each sample allows others to potentially assess copy number if it was not previously assessed. For instance, the publication of data only as plots of site frequency, which is common in the literature, does not meet these standards. To facilitate this, data reporting standards are needed, particularly for this level of data. Consequently, it is important to define what data should be minimally and optimally reportable (Table 2). There are well-developed public locations for the submission of raw sequencing data, such as the National Center for Biotechnology Information’s Short Read Archive and the European Nucleotide Archive. At a minimum, initial variant data should be presented as machine-readable tables deposited as supplemental, publicly available data. The selection of storage for variant data is more variable, and newer, flexible centralized repositories for disparate data, such as Zenodo (zenodo.org), which is housed at European Organization for Nuclear Research (CERN), can provide storage for variant calls that are currently evolving. Individual project-based variant viewers have also been leveraged.^50^

### Certain aspects of metadata are critical for future use.

Data sharing must comply with local ethical board requirements, with all individual-level data shared in deidentified formats. Within these constraints, the most precise information allowable that complies with these regulations should be made available. First, the geographic location of sample collections should be as precise as possible to allow for accurate mapping, intervention deployment, and understanding of impacts.^25^^,^^51^ This may include jittered household-level data, but it may be limited to the health district of collection. Second, approximate dates of collection should be shared because seasonality is important for malaria transmission and temporal trends. It is expected that the prevalence of mutations may vary depending on when they are collected during the transmission season.^51^^–^^53^ Precise dates should not be shared, but month or season and year may be allowable. Third, demographics play an important role in determining malaria risk; therefore, age and sex should be considered essential components.^54^^,^^55^ Fourth, human mobility is a critical aspect for studying malaria importation and migration of parasites; therefore, travel data should be included when available.^56^ Fifth, treatment history (e.g., pretreatment sample versus post-treatment sample) is critical for understanding the data, particularly in TESs in which recurrent parasitemia is occurring and post-treatment samples are not representative of the background frequency of mutations because of selective pressure within individuals, making resistant parasitemia more likely in these samples. Lastly, the clinical status of the individuals (e.g., whether the study involves clinical cases or asymptomatic cases) should be reported. Similar to variant calls, public repositories like Zenodo provide flexible options for metadata storage. Other options exist, such as university-run data repositories, exemplified by the University of North Carolina Dataverse (https://dataverse.unc.edu/), or within publications themselves.

### Multiple technical aspects need to be reported to assess the quality of variant data and samples.

First, absolute values of sequencing reads (or the depth) are needed to estimate within-sample allele frequencies for mixed infections. Because of malaria infections by multiple parasite strains, the observed prevalence of a resistance marker within a population depends on both its frequency and the average number of mixed infections in the population. Mixed infections are often not reported or estimated. Therefore, absolute values of sequencing reads allow for more suitable comparisons between studies conducted in different transmission intensities. These underlying values can also be important in facilitating quality reassessment, particularly in large studies where false positives become more likely because of the high number of tests. For example, if a variant allele occurs only at a low frequency across a large number of samples, this raises concern for false-positive calls because of a sequencing error or contamination. The read depth also allows for better filtering for secondary analyses that may be prone to different levels of allowable error. Lastly, this detail allows for the assessment of sample duplication in studies.

Robust controls should be reported for all MMS, preferably standardized panels that can be used across laboratories, such as those being developed by the WHO and others.^57^^,^^58^ In addition, quality control should be conducted by repeating 5–10% of samples and reporting the genotype concordance. The use of replicate samples can improve data quality and interpretability in two ways: 1) helping to assess sample quality and 2) improving within-sample allele frequency estimates. In the first case, replicates are necessary because the quality of samples can vary significantly depending on their source. Although some studies may have detailed chains of custody with well-documented storage conditions, many others have less reliable information. For example, large national surveys, such as demographic health surveys, involve dried blood spots that pass through multiple hands, are stored under unclear conditions in the field, and have been used for other assays before being made available for MMS.^25^^,^^26^ Many clinic-based MMS systems or TESs leverage health site care workers to collect samples and store them with minimal ongoing supervision. In the second case, the uncertainty of within-sample allele frequency estimates due to jackpotting (overamplification of certain alleles due to chance or biases) provides a strong argument for replicating all samples when the accurate measurement of allele variant absence, presence, or frequency is essential to the question (e.g., TESs) because 5–10% replicates indicate what percentage of samples may go awry but not which ones.^59^

Specific technology and kits used for sequencing, as well as details of filtering and data processing, are needed. When research methods are described only in piecemeal fashion—listing programs and key parameters—important details are often omitted, making it difficult to reproduce or validate findings. Sharing the exact code, such as through GitHub (GitHub Inc., San Francisco, CA) or another repository, is a step forward; however, challenges can still arise if others are not using the same platform or environment. Installing and running the software for a pipeline is often challenging. Issues around bioinformatics code and genomics data analysis tool usability and availability have been recently reviewed using defined software standards criteria,^44^ and work has been conducted to improve the usability of software for MMS.^60^ Beyond this, leveraging containers that can encapsulate and make the entire analysis pipeline for a dataset or manuscript portable, such as Docker and Singularity, are even more optimal, as well as newer shared cloud environments, such as Code Ocean’s web-browser-based “compute capsules” (Code Ocean, New York, NY), adopted by Nature Journals, which allow code to be run in an encapsulated environment with proper version control from a web browser (https://codeocean.com/resources/nature-partnership).^41^

Finally, data are often used across multiple studies. In this case, denoting where else the data have already been presented and made publicly available in the data table is essential. There are instances in which the same data used in multiple publications are entered into meta-analyses multiple times, leading to bias.^61^ Subsequent publications should be required to include standalone supplemental data for the new sequencing data generated by the study, as well as a reference to the previous reporting of the data, including PubMed identifier or other identifiers, and delineate the extent of sample or data reuse. This can also help merge data (e.g., variant data for different genes published separately) and is a good practice for peer review of individual reports, making it clear when data or samples from previous work are being reused. Ultimately, reporting of individual-level data would guard against duplication, given the ability to have unique identifiers per infected sample that transcend individual publications.

### Notably, nontargeted approaches are also leveraged in MMS.

In the present study, the focus has primarily been on reporting targeted sequencing data because it is the most widely generated data in MMS. However, certain aspects of MMS require more detailed data that are best generated using WGS. In particular, studying flanking regions of important genes to understand their origin and spread from a specific origin can provide potentially useful information. Deciding on the best approaches and analysis methods for this is complicated and beyond the scope of the current work. The size of the haplotype block may vary based on the type and age of the selective sweep involved. Currently, WGS represents only a fraction of the data generated for MMS. However, in general, similar principles of individual-level metadata and genetic data in public repositories would still apply.

### Although the goal of sharing individual-level data is widely supported, several challenges must be addressed.

First, researchers often hesitate to share data because of concerns about being “scooped” on potential analyses. To mitigate this issue, mechanisms should be established to ensure that data generators have priority in publishing planned analyses.^62^ Second, not all study authors obtain consent from participants for public sharing of individual-level data, raising ethical and privacy concerns, including the risk of identifiability. To protect participants, shared data should adhere to appropriate consent protocols and be presented in a manner that minimizes re-identification risks, especially when accurate geolocation and demographic information are included with samples. Privacy protections can be further strengthened through the use of secure data repositories with tiered access, where only approved researchers can access specific datasets under predefined conditions.^63^ Third, data colonialism remains a significant concern. Achieving equitable data sharing requires a fundamental shift in research culture, alongside financial and policy support from funders, research institutions, journals, and governments.^62^^,^^64^ This involves integrating equity into data-sharing policies, recognizing all intellectual contributions to research, and aligning academic recognition with data-sharing mandates to ensure appropriate rewards for meta-analyses, data sharing, and capacity-building efforts. Investments in human resources, infrastructure, and collaborative networks are also necessary to strengthen data curation and secondary data analysis capacity in low- and middle-income countries, as well as to develop sustainable and inclusive platforms for complex data integration and analysis. Furthermore, concerns about commercialization and benefit sharing must be addressed. Publicly available data may be leveraged by companies to develop profitable products and services without direct benefits to the communities that provided the data. Ensuring that individuals and communities share in the benefits of research outcomes is essential; mechanisms such as licensing agreements for data use can help address these issues.

### Addressing the question of how to represent genomic data.

In the present study, the focus has been on “what” data should be reported and “why,” rather than “how.” There are multiple potential data formats available for reporting data, particularly genomic data. For whole-genome data or SNP data, standard formats, such as variant call format (VCF), are an excellent choice. Targeted sequencing also often employs these same formats, but data are lost for nonvariant regions and haplotypic linkage in mixed infections. Better formats for reuse include genomic variant call format (GVCFs; or more compressed ReblockGVCFs) that retain information on nonvariant regions. Ultimately, preserving microhaplotypes, which are the most direct representation of error-corrected sequenced PCR products, leads to minimal loss of information compared with the raw sequence data. Drawing from malaria research, one format, the portable microhaplotype format (portable microhaplotype object [PMO]; https://plasmogenepi.github.io/PMO_Docs/), is an attractive lossless (full microhaplotypes) intermediate format under active development to promote the harmonization of analysis pipelines with negligible raw data loss. It is also designed to capture, in a systematic manner, appropriate metadata relevant to malaria epidemiology and MMS. Longer-term standardization by the community of robust, well-defined epidemiological vocabulary will be useful. Because the PMO epidemiological metadata is potentially a standalone format, it could be adopted for WGS and other next-generation sequencing pipelines and not solely limited to projects that generate microhaplotypes. Other data standards may be required at various points for downstream analyses; for example, the spatial-temporal aggregate variant encoding (STAVE) (https://github.com/mrc-ide/STAVE) package is designed to provide a flexible and convenient format for site and temporal aggregate genotype data that can be used in prevalence mapping. Importantly, these formats and related tools can increase the speed of analysis within laboratories and could particularly aid programs working with multiple laboratories in integrating their own data rather than attempting to merge unstandardized data across studies. These formats have the potential to be used beyond malaria research and for targeted sequencing across multiple organisms, forming the backbone of a unified data analysis ecosystem for targeted sequencing.

The malaria community must mount a coordinated response to the emerging threats of antimalarial drug and diagnostic resistance in Africa. The power of data sharing can be harnessed to provide critical insights into parasite biology and the drivers of resistance that extend well beyond what the authors of individual studies can achieve alone. Given that parasites do not respect political borders, the malaria community must also work across borders (and study boundaries). Striving for the highest-quality malaria MMS data and reporting is a critical step toward overcoming challenges faced by the African region and improving public health.
